# Enhancing Growth in *Vigna radiata* through the Inhibition of Charcoal Rot Disease: A Strategic Approach Using Plant Growth-Promoting Rhizobacteria

**DOI:** 10.3390/microorganisms12091852

**Published:** 2024-09-06

**Authors:** Imran Khan, Sahar Ghulam Mohyuddin, Sohail, Shah Zaman, Muhammad Qadir, Juxian Guo, Guihua Li

**Affiliations:** 1 Guangdong Key Laboratory for New Technology Research of Vegetables, Vegetable Research Institute, Guangdong Academy of Agricultural Sciences, Guangzhou 510642, China; dh18006@yzu.edu.cn; 2 College of Animal Science and Technology, Yangzhou University, Yangzhou 225009, China; 3 College of Bioscience and Biotechnology, Yangzhou University, Yangzhou 225009, China; 4 Department of Botany, University of Malakand KPK, Chakdara 18800, Pakistan; 5 Department of Botany, Abdul Wali Khan University Mardan, Mardan 23200, Pakistan

**Keywords:** biocontrol agents, biofertilizers, *Macrophomina phaseolina*, PGPR, *Vigna radiata* L.

## Abstract

*Macrophomina phaseolina* is a vital seed and soil-borne phytopathogen responsible for substantial crop yield losses. Although various methods exist for managing soil-borne pathogens, such as agronomic practices, chemical treatments, and varietal tolerance, biological control utilizing plant growth-promoting rhizobacteria (PGPR) or their secondary metabolites presents promising avenues. In this study, a screening of 150 isolates from the rhizosphere of *Vigna radiata* L. was conducted to identify strains capable of promoting host growth and controlling charcoal rot disease. Among the tested isolates, only 15 strains demonstrated the ability to produce plant growth-related metabolites, including indole acetic acid, hydrogen cyanide, ammonia, and lytic enzymes, and solubilize inorganic phosphate. Subsequently, these potent strains were evaluated for their antifungal activity against *Macrophomina phaseolina* in vitro. Three strains, namely MRP-7 (58% growth inhibition), MRP-12 (55% growth inhibition), and MRP-8 (44% growth inhibition), exhibited the highest percent growth inhibition (PGI.). Furthermore, a pot experiment demonstrated that the selected strains acted as effective growth promoters and ROS (reactive oxygen species) scavengers, and served as potential biocontrol agents, significantly reducing the incidence of charcoal rot disease and improving various agronomic attributes of the host plant. These findings highlight the potential of these strains to be utilized as biofertilizers and biocontrol agents for sustainable agricultural practices.

## 1. Introduction

*Vigna radiata*, commonly known as mung bean, is an essential leguminous crop considered a major source of protein, vitamins, and minerals [1]. However, the yield of mung beans is reduced due to various factors, with fungal diseases being of major importance. Among fungi, *Macrophomina phaseolina* is the main cause that lowers the yield and production of mung beans [2,3]. While there are about 500 different species of *M. phaseolina*, most of them do not show pesticide resistance [4]. However, the long-term use of pesticides has harmful impacts on soil properties, the environment, and host plants. The indiscriminate application of pesticides, including bactericides, fungicides, and insecticides, eliminates plant pathogens or disease vectors [5]. However, the undesirable effect of these chemicals and their degradation products may pose serious threats to the environment and human beings, leading researchers and growers to explore new and eco-friendly modes of disease control [6].

Alternative approaches, such as plant growth-promoting rhizobacteria (PGPR) as biocontrol agents, have been considered successful and are progressively being introduced in the field [7]. PGPR increases plant growth directly and indirectly and decreases the proliferation of diseases in the plant system by various pathways, including the synthesis of antimicrobial metabolites, volatile compounds, induced systemic resistance (ISR), etc. [8]. Such protection mechanisms can contribute to major changes in plants’ structural and functional changes that lead to pathogen resistance. PGPR can influence plant growth either directly or indirectly [9].

The accurate process by which bacterial strains increase the growth of plants is not completely known. However, it is considered to be due to their capability to generate or change the number of growth promoters, such as auxin, cytokinin, ethylene, gibberellic acid, phosphate, and other nutrient solubilization [10], antifungal activity against disease-causing microbes by siderophore production [11], production of hydrogen cyanide [12], antibiotics production [13], and atmospheric fixation of nitrogen [14].

Therefore, the current study aimed to (i) isolate potent strains from the rhizosphere of *Vigna radiata*, (ii) evaluate their potential for plant growth promotion, and (iii) investigate their role in the suppression of charcoal rot disease caused by infection of *M. phaseolina*. This research paper presents the study’s findings to explore the potential of rhizobacteria isolated from *Vigna radiata* as a potential plant growth promoter and biocontrol agent against *M. phaseolina* in vitro.

## 2. Materials and Methods

### 2.1. Isolation and Purification of PGPR

The serial dilution method was used to isolate plant growth-promoting rhizobacteria (PGPR) from the rhizosphere of mung bean (*Vigna radiata* L.). Following the protocol, 1 mL of rhizosphere soil solution was mixed with 9 mL of autoclaved distilled water and thoroughly shaken. Serial dilutions were performed up to 10^−6^ dilutions. From each dilution, 100 µL was spread onto Luria–Bertani (LB) agar plates. The plates were then incubated at 28 °C for 24 to 48 h. Morphologically distinct colonies observed on the agar plates were further purified to obtain axenic cultures. These cultures were stored at low temperatures for subsequent experimental use.

### 2.2. Morphology and Gram Staining of Selected Isolates

The selected strains were evaluated for morphological features, including colony shape, colony color, colony margins, elevation, and opacity. For Gram staining, a smear of the bacterial culture was prepared. The smear was first treated with crystal violet for 30 s and then rinsed with distilled water. Next, Gram iodine was applied for 30 s and decolorized with 70% ethanol. The smear was washed with dH2O and then stained with safranin. Finally, the smears were visualized using a light microscope (Eclipse Ci-L, Nikon Instruments Inc., Melville, NY, USA) [15].

### 2.3. Biochemical Profiling

#### 2.3.1. Detection and Quantification of Indole Acetic Acid

The production of indole acetic acid (IAA) was assessed using the Kovács reagent method. The presence of a red ring formation indicated the presence of IAA, while a green ring formation represented its absence [16]. For quantification, the concentration of IAA in the culture supernatant of the selected strain was determined using the Salkowski reagent and a spectrophotometer (PerkinElmer Lambda 25 double-beam spectrophotometer) at 540 nm as followed by Qadir et al. [17].

#### 2.3.2. Hydrogen Cyanide (H.C.N.) and Ammonia (NH_3)_ Production

HCN Assessment: To assess hydrogen cyanide (HCN) production, the strains were inoculated in a broth medium containing 4.4 g of glycine. Whatman’s filter paper no. 1 was immersed in a mixture of 2% sodium carbonate and 0.5% picric acid solution, then placed on the plate lid. The plates were sealed airtight with parafilm and incubated at 30 °C for 4 days. A change in the color of the filter paper from yellow to orange or brown indicated hydrogen cyanide production [18]. 

Ammonia Production: To assess ammonia production, 10 mL of freshly prepared peptone water was inoculated with selected bacterial isolates and incubated at 28 ± 2 °C for 48–72 h. After incubation, adding 1 mL of Nesseler’s reagent resulted in the formation of an orange-to-yellow color, indicating the presence of ammonia [19]. 

### 2.4. Enzyme Production 

Catalase Activity: A drop of 3% H_2_O_2_ was directly placed on a glass slide containing the bacterial inoculum to assess catalase activity. The presence of rapid gas bubble production or effervescence indicated catalase activity [20].

#### 2.4.1. Protease Activity

For protease activity evaluation, the selected isolates were inoculated on plates prepared with skimmed milk agar medium and incubated at 35 °C for 48 h. The formation of clear zones in the vicinity of the bacterial colonies indicated protease activity [21]. 

#### 2.4.2. Amylase Activity

To determine amylase activity, the isolates were streaked on respective media plates and incubated at 37 °C for 48 h. Subsequently, the plates were flooded with a freshly prepared 1% iodine solution. The formation of clear zones or hydrolysis zones around the bacterial culture indicated the presence of amylases [22].

#### 2.4.3. Pectinase Activity

The selected rhizobacteria were inoculated on respective media plates and incubated at 35 °C for 24 h to assess pectinase enzyme production. The confirmation of pectinase activity was observed by the formation of clear zones around the bacterial colonies [23].

### 2.5. Phosphate Solubilization Ability

To evaluate the phosphate solubilization potential, all isolates were cultivated on Pikovskaya’s agar medium [24]. A pin-point inoculation was made on the agar plates, and the plates were then incubated at 28 °C for 7 days. The development of a halo zone around the bacterial colonies indicated their capability for phosphate solubilization. The extent of phosphate solubilization by the strains was calculated using the following equation.

$$Solubilization\;Index=\frac{Colony\;Diameter+Halo\;zone\;Diameter}{Colony\;Diameter}$$

### 2.6. In Vitro Antagonistic Activity

To assess the antagonistic activity using the dual-culture method, the bacterial isolates were evaluated for their ability to inhibit *Macrophomina phaseolina* on Potato Dextrose Agar (PDA) plates [25]. A loopful of bacterial cultures, which were 2 days old, were individually inoculated at one end of the plate, maintaining a distance of 1 cm from the margin. A 6 mm disk of the fungal pathogen culture was placed on the opposite side of the plate. The plates were then incubated at 28 °C for 4–5 days. Plates without any bacterial antagonists were used as the control. The activity against the fungus was observed by assessing the bacteria’s inhibition of fungal mycelial growth. The antifungal activities of each bacterial strain were assessed in replicates. The percentage of inhibition was calculated using the following formula:

$$Percent\;Growth\;Inhibition\left(PGI\right)=\frac{Fungal\;growth}{Control\;Growth}\times 100$$

### 2.7. Pot Experiment

Soil Preparation: A soil mixture was prepared by combining sand, field soil, and manure in a ratio of 1:2:1. Diammonium phosphate (DAP) was added to the soil at a rate of 60 kg hec^−1^ to provide essential nutrients for plant growth.

#### 2.7.1. Selection of Pot Experiment Factors

The experiment included control plants with no fungus and PGPR, plants treated with the pathogen, and plants treated with both the pathogen and PGPR. These factors were arranged in a factorial combination to evaluate their effects on plant growth and biocontrol potential.

#### 2.7.2. Selection of Mung Bean Variety

The mung bean variety “Azzri-06” was obtained from the Plants Genomics Research Institute (PGRI) and the National Agricultural Research Centre (NARC), Islamabad, Pakistan. The experiment was performed at the NARC during the 2022 season. The mung bean variety “Azzri-06” is a high-yielding cultivar developed in Pakistan, specifically bred for its adaptability to the country’s climatic conditions. It is known for its resistance to common diseases such as mung bean yellow mosaic virus (MYMV) and powdery mildew, making it a reliable choice for farmers.

#### 2.7.3. Seed Preparation

Mung bean seeds were surface sterilized using 0.1% HgCl_2_ solution and rinsed three times with distilled water. A sugar solution was prepared by dissolving 10 g of sugar in 50 mL of distilled water to serve as an adhesive material. The surface-sterilized seeds were immersed in the sugar solution for 20 min to ensure proper adhesion of the selected PGPR strains. Seeds assigned to the control group were only washed with water.

#### 2.7.4. Planting and Treatment Application

Five seeds were planted in each pot containing 10 kg of the prepared soil. The pots were labeled according to the treatment combinations. The selected PGPR strains were coated onto the seeds in the respective treatment groups, while the control seeds were left untreated.

#### 2.7.5. Growth Period

The pots were placed in a controlled environment with suitable temperature, light, and humidity conditions. The plants were allowed to grow for 60 days after applying the treatments.

#### 2.7.6. Harvest and Data Collection

After 60 days, the plants were harvested, and various growth attributes were recorded. These included measurements of the root and shoot length, fresh and dry weight of the plants, the number of pods produced per plant, and the number of nodules formed on the roots.

### 2.8. Chlorophyll Determination

For the determination of the total chlorophyll contents, approximately 0.1 g of the plant material was ground in 80% acetone. After fully homogenizing the plant tissue, the samples were centrifuged at 10,000 rpm to remove the plant debris. The resultant supernatant containing chlorophyll contents was estimated using a double-beam spectrophotometer at 480, 645, and 663 nm. The chlorophyll contents were measured as mg/g of fresh plant materials [26].

### 2.9. Plant Antioxidant Determination

#### 2.9.1. Peroxidases

For extraction of the peroxidases, different parts of the plant were collected and preserved at −80 °C. The extraction buffer was first made by mixing 50 mM potassium phosphate buffer (pH 7.0), 1 mM ethylenediaminetetraacetic acid (EDTA), 1% polyvinylpyrrolidone (PVP), and 1% polyethylene glycol (PEG). The mixture was then kept chilled. Next, utilizing a mortar and pestle and liquid nitrogen to grind the plant material into a fine powder, quick processing was ensured to avoid thawing. Subsequently, at a ratio of around 1:2, the powdered plant material and the pre-chilled extraction buffer were combined, and the combination was completely homogenized on ice. Following homogenization, to separate the supernatant containing the extracted peroxidase from the cellular debris, the extract was centrifuged at a low temperature and high speed. After being carefully transferred to a new, pre-chilled microcentrifuge tube, the peroxidase-enriched supernatant was advised to be stored at −80 °C for long-term preservation [27].

#### 2.9.2. Estimation of POD

Guaiacol was used as a substrate for dehydrogenation in order to carefully measure peroxidase activity, a crucial sign of oxidative stress and plant defense systems, following the established protocol by Wang, Qiu, Yang, Huang, Dai, Zhang, and Zou [27]. Using a phosphate buffer (3 mL of 0.1 M, pH 7) to ensure ideal conditions for enzyme stability and activity, the extraction of peroxidase was carried out precisely. A 1 mL phosphate buffer was used to crush 1 g of fresh leaves, and the mixture was centrifuged for 20 min at 8064 rcf and 5 °C. The supernatant was collected carefully for enzyme extraction. For enzyme activity, the peroxidase assay was carefully formulated. It included 0.1 mL supernatant, 0.1 M phosphate buffer with 3 mL, 0.05 mL of 20 millimolar guaiacol solution, and 0.03 millimolar of 12.3 mM or 0.04% hydrogen peroxide solution. In the presence of hydrogen peroxides, guaiacol may be effectively converted to peroxidase. The optical density of 436 nm of the reaction mixture was measured with a predetermined time period (*t* = 0.1). The plant’s biochemical dynamics in response to external stimuli were furthered by applying a robust computational framework to the gathered data and quantifying peroxidase activity using an equation that has been validated by science.

$$Enzyme\;activity=\left(\frac{500}{∆t}\right)\times \left(\frac{1}{1000}\right)\times \left(\frac{TV}{VU}\right)\times \left(\frac{1}{fwt}\right)$$

where

Δt = change in time; TV = total volume; VU = volume used; f wt = fresh weight (g).

#### 2.9.3. Superoxide Dismutase 

##### Extraction

Plant material was used for the estimation of enzyme activity, and superoxide dismutase (SOD) was a carefully considered approach. In the process, the extraction buffer was made by mixing a protease inhibitor (Roche, Mannheim, Germany) with 50 mM KH2PO4 (pH 7.8), 100 µM EDTA, 1% Triton X-100, 2% polyvinylpyrrolidone, and other ingredients. An amount of 0.1 g of fresh plant material in a buffer with precisely ground fresh plant material (0.1 g) sonicated for 2 × 30 s at A = 30 at 4 °C using a LeelaSonic-50 model sonicator was used and filtered through polycarbonate filters (Osmonics, South Miami, FL, USA) featuring 2.0 µm pore diameters. The filtrate was centrifuged for 20 min at 5600 rcf at 4 °C, following the method described by Qadir et al. [28]. For SOD measurement, supernatants were utilized.

##### Estimation

The techniques used by Qadir et al. [28] were followed for SOD activity. To obtain a final volume of 3 mL, the reaction mixture was supplemented with 50 millimolar phosphate buffer (pH 7.6), 100 micromolar EDTA, 50 mM sodium carbonate (Na_2_CO_3_), 50 μM NBT, 10 μM riboflavin, 12 mM L methionine, and 100 μL in the plant sample extract. Against the blank. For initiation of reaction, the sample was kept for fifteen minutes. At 560 nm, OD was measured. The combination’s optical density was then measured at 560 nm. One unit (U) of SOD is required to stop half of the photochemical degradation of NBT; the measurement of SOD activity provides a solid foundation for scientific comparisons and investigations of antioxidant enzyme activity in plant samples.

#### 2.9.4. Ascorbate Peroxidase 

To maintain enzyme activity, fresh plant material was obtained, and then quickly frozen in liquid nitrogen. Next, using a mortar and pestle that had been previously refrigerated with liquid nitrogen, about 2 g of the frozen plant material was pounded into a fine powder. A tube that had been cooled beforehand received the resultant plant powder. An amount of 50 mM phosphate buffer (pH 7.0) in a cold buffer was used as the extraction buffer. Amounts of 1 mM ascorbic acid, 0.1 mM ethylenediaminetetraacetic acid (EDTA), and 1% polyvinylpyrrolidone (PVP) were prepared and kept on ice. In the powdered plant, the buffer was added (about 5 mL per gram of plant material) and ground with a motor and pestle. An optimum temperature was required for the enzyme activity. The mixture was homogenized and centrifuged at 4 °C for 15 min at 10,000 rpm. The resulting supernatant containing ascorbate peroxidase (APX) and other soluble proteins was kept at a low temperature for further use in the analysis of the selected enzymes [29,30].

#### 2.9.5. Estimation of APX

Tripathi et al.’s [31] methods were followed for the ascorbic peroxidase activity with a few minor adjustments. Plant extract (1 mg/mL in 0.1 M phosphate buffer, pH 7) in 0.6 mL 3 mL of a reaction mixture was made. There were amounts of 1.5 mL of ascorbic acid, 50 mL of phosphate buffer (pH 7.0), and 0.1 mL of hydrogen peroxides. The absorbance decreased over time at 290 nm. A total of 0.1 absorbance of one unit (U) was the change in per minute per milligram of protein [30]. 

### 2.10. Statistical Analysis

The experiments were repeated three times to ensure reliable and robust results. Each replication was conducted under the same conditions and with the same materials. The data obtained from the factorial experiment, which included control, pathogen-treated, and bacterial-inoculated conditions, were grouped accordingly for further analysis. ANOVA was performed on the grouped data using SPSS software (IBM SPSS Statistics 21). ANOVA is a statistical technique determining the significance of differences between groups or conditions. It helps identify if there are significant effects of pathogen treatment and rhizobacterial inoculation on the measured variables. After conducting ANOVA, Duncan’s Multiple Range Test (DMRT) was performed to compare the means of different treatments and identify significant differences between them. Graphs illustrating the results were plotted using Graph Pad Prism (Version 5.03) software. Graph Pad Prism is a well-known software tool for graphing and analyzing scientific data.

## 3. Results

### 3.1. Morphological Characterization of a Selected Strain

After incubation for 24 h, 150 morphologically different bacteria were selected. For screening the desired metabolite production, 15 isolates were further processed to obtain axenic cultures. These isolates exhibited variations in colony color, ranging from off-white to pure white, lemon, and red. The colony forms were predominantly circular, although irregular and punctiform forms were also observed. The elevations of the isolates varied, with most being flat, raised, or umbonate. Colony margins were predominantly observed as entire, lobate, or erose. The colonies displayed a range of transparency, some being translucent and others opaque. Microscopic observation revealed that six of the fifteen strains showed Gram-negative characteristics, while the remaining strains were Gram-positive (Table 1).

### 3.2. Biochemical Charactiristics of the Selected Isolates

#### 3.2.1. IAA, HCN, Ammonia (NH_3_) Production and Phosphate Solubilization

**Figure 1:**
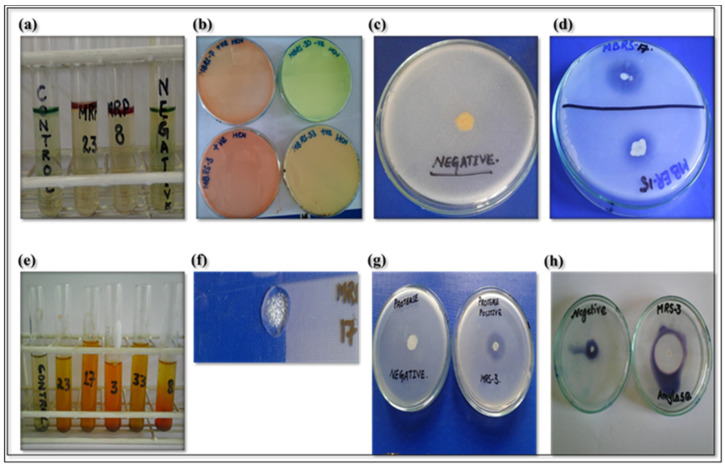
Screening of selected strain for (**a**) IAA, (**b**) HCN, (**c**,**d**) phosphate solubilization, (**e**) ammonia, (**f**) catalase, (**g**) protease, and (**h**) amylase production.

Among the metabolites produced by the isolates, phytohormones play a significant role in promoting plant growth and aiding in stress acclimation. The selected bacterial isolates were tested for indole-3-acetic acid (IAA) production (Table 2). Out of the selected isolates, only MRP-8 and MRP-23 were found to produce IAA, with concentrations of 16.06 µg mL-1 and 9.7 µg mL-1, respectively (Figure 1). In addition to IAA production, MRP-7, MRP-8, MRP-12, MER-15, MRS-17, and MRS-33 exhibited the ability to produce hydrogen cyanide (HCN), while all strains showed potential for ammonia production (Table 2 and Figure 1).

Nine out of the fifteen isolates demonstrated the capability to solubilize inorganic phosphate, as indicated by the formation of a transparent halo zone around the bacterial colonies (Table 2). Strains MRP-8, MRS-17, and MER-15 exhibited the highest phosphate solubilization activity, with zone sizes of 1.1 mm and 1 mm, respectively. The solubilization index ranged from 3.16 to 2.56, with MRS-17, MRP-8, and MER-15 showing the highest solubilization index.

#### 3.2.2. Lytic Enzyme Generating Test

The selected isolates were assessed for producing various lytic enzymes, including catalase, amylase, protease, and pectinase (Table 3 and Figure 1). All 15 selected strains showed positive catalase activity, except for MRS-35. Regarding protease production, all strains tested positive except for MRP-34. MRS-20, MRS-29, and MRP-8 exhibited clear zone formation with diameters greater than 2 mm. Regarding amylase production, 11 strains displayed positive results, with MRS-29 and MRP-7 showing the largest zone formations, measuring 2 mm and 1.8 mm, respectively. Additionally, all 15 strains demonstrated positive results for the pectinase test, with most strains forming clear zones larger than 2 mm in diameter.

### 3.3. Dual-Culture Test

**Figure 2:**
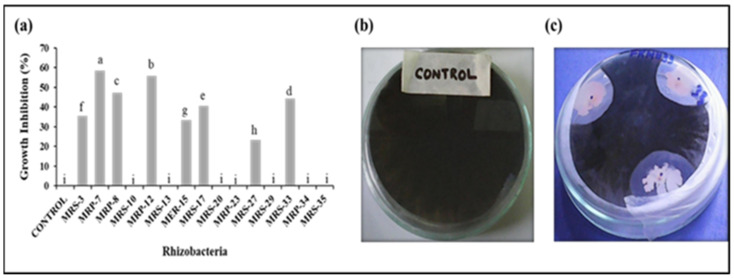
Screening of selected strains for (**a**) % inhibition of fungal strain, (**b**) fungal culture, and (**c**) dual culture of potent strains with fungus.

The antifungal activity of the selected strains was evaluated by conducting dual cultures with the fungal pathogen *Macrophomina phaseolina* (Figure 2a–c, Table 4). Out of the tested strains, eight exhibited antagonistic activity, as evidenced by the formation of zones of varying diameters. Notably, the bacterial strains MRP-7, MRP-8, MRP-12, MRS-17, and MRP-33 displayed higher antagonistic potential under in vitro conditions, with percent growth inhibition ranging from 24% to 59%.

### 3.4. Pot Experiment Results

**Figure 3:**
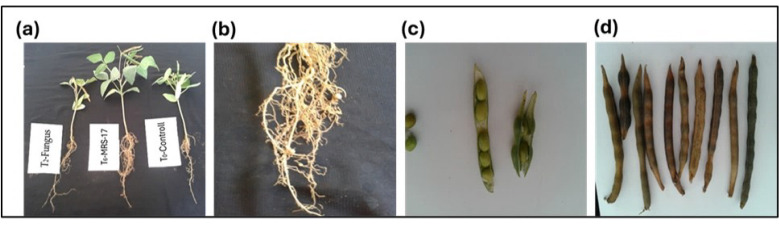
Effect of PGPR on (**a**) root shoot length, (**b**) No. of nodules, (**c**) seeds per pod, and (**d**) No. of pods per plant on the host plants.

**Figure 4:**
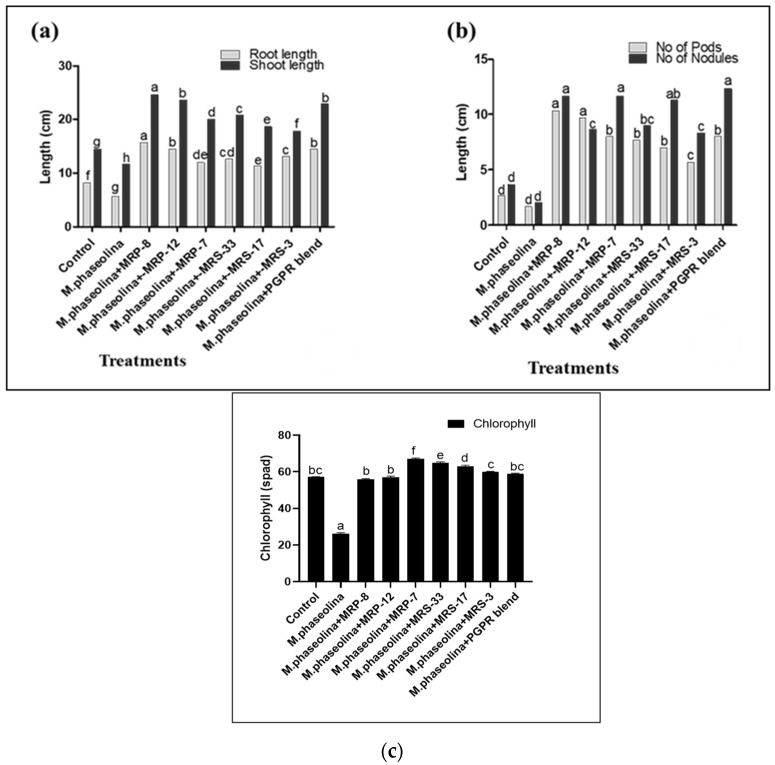
Effect of PGPR and *M. phaseolina* on (**a**) root and shoot length, (**b**) No. of seeds and pods of the host. (**c**) the total chlorophyll contents. Data are mean of replicate with SE± and letters represent the significant difference (*p* < 0.05).

The pot experiment results demonstrated that all selected strains exhibited the ability to inhibit the development of charcoal rot disease in the roots of mung beans, surpassing the control group (fungus). Furthermore, the strains significantly improved various growth parameters, including the root and shoot length, number of pods, and number of nodules (Table 5 and Figure 3 and Figure 4). Notably, strain MRP-8 displayed the most prominent effects, enhancing the shoot length up to 27.66 cm, root length up to 13.66 cm, and increasing the number of pods to 11 and the number of nodules to 3 compared to all other treatments and the control group. Conversely, strain MRS-3 exhibited relatively minimal effects. Moreover, the combination of selected strains demonstrated similar improved results, further highlighting their synergistic potential. In the case of the chlorophyll contents, higher reductions were recorded in the total chlorophyll contents at the onset of the pathogen interactions. In the PGPR inoculation status, higher chlorophyll contents were recorded in the case of MRP-7, MRS-33, 17, and MRS-3. The MRP-8 and 12 recovered the chlorophyll contents comparable to control plants.

**Figure 5:**
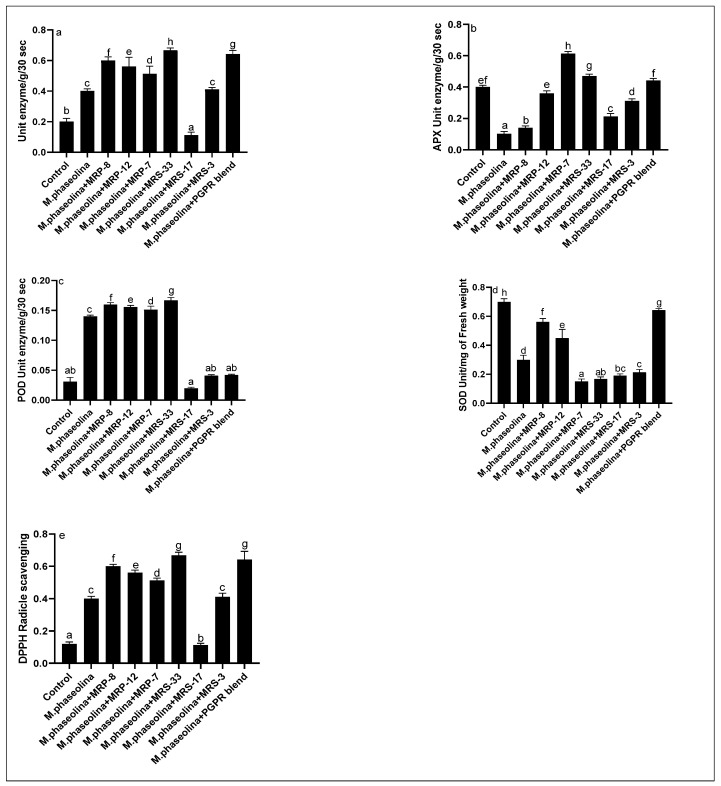
Effect of PGPR and *M. phaseolina* on (**a**) catalases, (**b**) ascorbate peroxidases, (**c**) peroxidases, (**d**) superoxide dismutase, and (**e**) DPPH radical scavenging activity of the host. Data are mean of replicate with SE± and letters represent the significant difference (*p* < 0.05).

In the case of the antioxidant’s activities, the catalase showed an increase with the increase in the pathogen attack. A further increase was recorded in all PGPR inoculation both in the separate and blend conditions. However, a reduction was recorded in the case of the MRS-17, showing a reduction of approximately half as compared to the control plants (Figure 5a). Contrasting results were recorded in the APX production, both in the pathogen inoculation and in most cases of PGPR inoculation, showing a reduction as compared to the control plants (Figure 5b). Contrasting results were recorded in the case of MRP-7 and MRS-33, showing an incline trend. An abrupt uptrend was recorded in the case of peroxidase production (Figure 5c). An incline was recorded in the case of the pathogen inoculation to the host plants, showing an increase of double compared to the control plants. The increasing trend was followed by the PGPR as well, showing a higher production of the peroxidase enzymes. However, in the case of MRS-17, 3, and blend of all the strains, a decline was recorded in the peroxidase production (Figure 5d). Similarly, a decline was recorded in the case of the SOD production when the plants were inoculated with the pathogen strains. A similar decline trend was kept continuous by the PGPR as well, showing a lower SOD as compared to the control plants. A contrasting result was recorded compared to the SOD in the case of DPPH radical scavenging activity, and an increase was recorded in the case of the pathogen and PGPR as well, showing a multifold increase; however, a reduction was recorded in the case of MRS-17, showing a lower DPPH radical scavenging activity (Figure 5e).

## 4. Discussion

The increasing prevalence of infectious diseases, particularly those caused by drug-resistant bacteria and fungi, has led to a growing interest in natural products and the search for new antimicrobial drugs [32]. Plant growth-promoting rhizospheric microbes have emerged as promising candidates due to their potential to enhance host growth and secrete secondary metabolites that suppress various diseases while boosting host immunity [33]. These microbes employ different strategies for promoting host growth, including direct mechanisms involving the production of plant growth regulators and indirect mechanisms such as the suppression of pathogen growth [33,34].

In this study, a total of 150 rhizobacterial isolates were evaluated for their potential as growth-promoting and biocontrol agents against mung bean. Among these isolates, fifteen bacterial strains were selected based on their appearance, Gram stain reaction, and biochemical tests. Of the fifteen strains, six were Gram-negative and nine were Gram-positive. Six strains could produce hydrogen cyanide (H.C.N.), while MRS-3, MRP-8, MRS-13, MER-15, MRS-17, MRP-23, MRS-27, MRP-34, and MRS-35 exhibited phosphate solubilization ability [35]. Additionally, MRP-8 and MRP-23 were able to produce indole acetic acid (IAA), and all strains were capable of producing ammonia [36]. Moreover, all the strains exhibited lytic enzyme production, which aids in decomposing fungal cell walls and hyphae, thereby suppressing disease initiation and providing host-induced systemic resistance. These characteristics make these rhizobacterial isolates suitable for controlling fungal pathogens [37]. 

In the present study, eight strains demonstrated the ability to inhibit the growth of the fungal pathogen *Macrophomina phaseolina* in dual-culture assays, with four strains exhibiting a greater inhibition zone against the fungal pathogen. Phytohormones, particularly indole acetic acid (IAA), play a crucial role in promoting plant growth [38,39]. The strains capable of producing indole acetic acid (IAA), solubilizing the insoluble phosphate, and producing ammonia have the potential to boost plant growth and yield. Such potential makes them the foundation of developing potent biofertilizers, helping the plant to acquire the essential nutrients while lowering their dependency on synthetic fertilizers, thereby contributing to more sustainable agricultural practices. While plants naturally contain small amounts of IAA, rhizobacteria can also secrete IAA to support host plants under normal and stressful conditions [40,41,42]. In our study, strains MRP-8 and MRP-3 produced promising quantities of IAA, contributing to better host growth regarding plant height, root growth, and the number of pods and seeds per pod. The variability in IAA release among different strains may be attributed to the different requirements for precursors [43]. For instance, 66 bacterial strains produced varying amounts of IAA in the presence and absence of the precursor tryptophan. The strains also exhibited sufficient ammonia production and the ability to solubilize inorganic phosphate, facilitating host growth by making insoluble phosphorus available for plant uptake [44]. Furthermore, MRP-7 showed higher quantities of HCN release, followed by MRP-8, MRP-12, MER-15, MRS-17, and MRS-33, which helps the host by suppressing harmful microbes in the rhizosphere while promoting host growth [45].

Apart from their role in secreting plant growth-promoting secondary metabolites, the selected strains also demonstrated the ability to suppress charcoal rot disease through the production of various lytic enzymes, including proteases, amylases, and pectinases, which disintegrate the cell walls of the pathogen, inhibiting their growth [34]. The production of hydrogen cyanide (HCN) and lytic enzymes like proteases, amylases, and pectinases suppresses the proliferation of phytopathogens such as M. phaseolina, showing their potency as effective biopesticides. The use of such potent strains in agriculture will aid in sustainable crop management strategies, which in turn reduce the need for chemical pesticides, which usually have a severe negative impact on the diversity of the environment in an unbearable way, thus promoting a more ecologically sound approach to pest control. The results showed that six bacterial strains effectively suppressed charcoal rot disease, significantly improving the shoot length, root length, number of pods, and number of nodules in *Vigna radiata* [46]. Among the selected strains, MRP-8 exhibited the maximum increase in the shoot length, root length, number of pods, and number of nodules, followed by MRP-12, MRP-7, MRS-33, MRS-17, and the combined inoculation, which also yielded effective results compared to the control. The suppression of charcoal rot disease by the selected strains was likely attributed to HCN production and the secretion of lytic enzymes, including proteases, amylases, and pectinases [47]. Microbes producing proteases, amylases, and pectinases decompose organic matter, promote plant growth, and act as biocontrol agents against pathogens [34]. 

The investigation into antioxidant enzyme activities under varying conditions of pathogen attack and PGPR inoculation revealed nuanced responses within the plant–microbe interaction system. These findings provide valuable insights into the intricate dynamics of plant defense mechanisms and the potential modulatory role of PGPR strains.

Catalase activity exhibited a discernible response to pathogen attack, indicating the plant’s adaptive defense mechanism against oxidative stress. This observation aligns with the established literature on catalase as a pivotal enzyme in mitigating reactive oxygen species (ROS)-mediated damage [48]. The escalated catalase activity further substantiates the plant’s activation of antioxidant defenses in response to pathogenic threats. Notably, a notable augmentation in catalase activity was observed in both the separate and blended PGPR inoculation conditions [49,50]. This augmentation suggests a synergistic effect of PGPR strains on catalase induction, potentially implicating their role in bolstering the plant’s antioxidant defense system. Similar findings have been reported in studies highlighting the beneficial effects of specific PGPR strains on enhancing plant stress tolerance [51]. The contrasting results in APX production observed in this study warrant further investigation. The reduction in APX activity following both pathogen and PGPR inoculation may signify a complex interplay between the plant, pathogen, and PGPR strains [49]. Previous studies have indicated diverse responses of APX in different stress scenarios, underscoring the multifaceted nature of plant defense mechanisms. The reduction in APX activity in the presence of PGPR strains may imply specific signaling pathways or biochemical interactions that necessitate in-depth exploration [52]. It is essential to consider the potential crosstalk between signaling pathways involved in pathogen defense and those modulated by PGPR strains. The observed incline in enzyme activity for MRP-7 and MRS-33 strains highlights their potential as beneficial microbes in enhancing the plant’s antioxidant defense network. These strains may harbor specific mechanisms that stimulate the plant’s antioxidant machinery, warranting further molecular and biochemical investigations. The abrupt uptrend in peroxidase production upon pathogen inoculation underscores the pivotal role of this enzyme in combating oxidative stress induced by the pathogen [53]. This heightened response aligns with the established literature on peroxidase as a key player in the plant’s defense against pathogens. Additionally, the enhanced peroxidase production in the presence of PGPR strains signifies their positive impact on reinforcing the plant’s antioxidative capacity [54]. This corroborates studies that have emphasized the potential of PGPR strains in augmenting plant defense mechanisms. The distinctive response exhibited by the MRS-17 strain, characterized by a reduction in catalase activity and peroxidase production, warrants careful consideration. This strain may possess specific attributes that interact differently with the plant’s defense system [55]. Further characterization of MRS-17′s molecular components and potential signaling pathways is essential for elucidating this unique interaction. The decline in SOD production following pathogen inoculation suggests a potential suppression of the plant’s SOD defense system. This phenomenon may be attributed to the pathogen’s evasion strategies or interference with the plant’s redox signaling pathways. The analogous decline observed in PGPR inoculation implies that the PGPR strains in this study may not exert a substantial influence on SOD activity [54]. This highlights the need for further investigations into the specific mechanisms employed by PGPR strains in modulating SOD responses [56]. The contrasting results in DPPH radical scavenging activity, with an increase observed in both pathogen and PGPR inoculation, pose an intriguing aspect of this study. The multifaceted nature of plant–microbe interactions may underlie this observation, necessitating comprehensive metabolomic and proteomic analyses. The reduction in DPPH radical scavenging activity in the case of the MRS-17 strain highlights the strain-specific variations in antioxidative responses [57,58]. The recorded boost in the production of antioxidants in response to both pathogen attack and PGPR inoculation ensures that the selected strains play a pivotal role in boosting crop resilience to multiple biotic stress factors. By strengthening the plant’s innate defense mechanisms, these PGPR strains could help maintain crop productivity under adverse conditions, contributing to the stability and sustainability of agricultural systems. This finding underscores the importance of discerning the molecular basis of such interactions for targeted applications in plant health management.

## 5. Conclusions

In conclusion, this study identified potential bacterial strains in the rhizosphere of mung bean with multiple plant growth-enhancing characteristics, including HCN production, indole production, NH_3_ production, enzyme production, phosphate solubilization, and suppression of fungal pathogens. These findings highlight the importance of isolating effective plant growth-promoting rhizobacteria (PGPR) that exhibit antagonistic behavior against pathogens, thereby contributing to sustainable agriculture and environmental preservation. Further field studies are recommended to assess the effectiveness of these potential strains. If proven successful, these strains can be recommended as bio-inoculants for agriculture, promoting soil health and ensuring sustainable crop production.

## Figures and Tables

**Table 1:** Morphological and microscopic observation.

Name	Colony Color	Form	Elevation	Margin	Opacity	Shape	Gram Reaction
MRS-3	Off-white	Irregular	Umbonate	Lobate	Translucent	Coccus	+
MRP-7	Off-white	Circular	Umbonate	Entire	Transparent	Bacillus	-
MRP-8	Creamy	Punctiform	Flat	Entire	Translucent	Bacillus	-
MRS-10	Creamy	Circular	Flat	Entire	Opaque	Coccus	+
MRP-12	Off-white	Irregular	Umbonate	Lobate	Translucent	Bacillus	-
MER-15	Light orange	Circular	Raised	Entire	Translucent	Bacillus	-
MRS-17	Off-white	Circular	Flat	Entire	Translucent	Bacillus	-
MRS-20	Creamy	Punctiform	Flat	Entire	Translucent	Coccus	+
MRP-23	Red	Circular	Flat	Entire	Opaque	Coccus	+
MRS-27	Off-white	Punctiform	Raised	Entire	Translucent	Coccus	+
MRS-29	Lemon	Circular	Flat	Entire	Opaque	Coccus	+
MRS-33	White	Circular	Flat	Entire	Translucent	Bacillus	-
MRP-34	Off-white	Circular	Raised	Entire	Opaque	Coccus	+
MRS-13	Off-white	Circular	Flat	Erose	Translucent	Coccus	+
MRS-35	Off-white	Circular	Flat	Entire	Translucent	Coccus	+

Where, + represents Gram positive and - represent Gram negative bacteria.

**Table 2:** Detection and quantification of I.A.A., H.C.N., NH_3,_ and phosphate solubilization.

Strain	Indole Acetic Acid		HCN	NH_3_ Detection/Color	P Solubilization	
	Detection	Quantification			Test	SI
MRS-3	-	0.0 ± 0.0	-	+/Orange	+	1.5
MRP-7	-	0.0 ± 0.0	+++	+/Yellow	-	0.0
MRP-8	+	16.06 ± 0.21	+	+/Yellow	+	2.2
MRS-10	-	0.0 ± 0.0	-	+/Orange	-	0.0
MRP-12	-	0.0 ± 0.0	+	+/Yellow	-	0.0
MRS-13	-	0.0 ± 0.0	-	+/Faint yellow	+	1.2
MER-15	-	0.0 ± 0.0	+	+/Orange	+	2.2
MRS-17	-	0.0 ± 0.0	+	+/Orange	+	3.2
MRS-20	-	0.0 ± 0.0	-	+/Faint yellow	-	0.0
MRP-23	+	9.7 ± 0.11	-	+/Yellow	+	1.5
MRS-27	-	0.0 ± 0.0	-	+/Orange	+	1.1
MRS-29	-	0.0 ± 0.0	-	+/Faint yellow	-	0.0
MRS-33	-	0.0 ± 0.0	+	+/Yellow	-	0.0
MRP-34	-	0.0 ± 0.0	-	+/Yellow	+	1.4
MRS-35	-	0.0 ± 0.0	-	+/Orange	+	1.2

+ represent production of IAA, NH3 production, and solubilization of P; - represents inability to produce IAA, NH3, and solubilize P; + low HCN production; +++ high HCN production.

**Table 3:** Detection of lytic enzyme.

PGPR Strain	Catalase Test	Protease Test	Amylase Test	Pectinase Test
MRS-3	+	+	+	++
MRP-7	+	+	++	++
MRP-8	+	+++	++	+++
MRS-10	+	+	++	+++
MRP-12	+	+	+	+++
MRS-13	+	+	+	+++
MER-15	+	+	-	+
MRS-17	+	++	-	++
MRS-20	+	+++	++	++
MRP-23	+	++	-	++
MRS-27	+	++	+	++
MRS-29	++	+++	+++	+++
MRS-33	+	+	++	++
MRP-34	+	-	-	++
MRS-35	-	++	+	+++

(**a**) Catalase test: - represents the absence of the activity. + represents normal and ++ represents two-fold higher enzymatic activity; (**b**) protease, amylase, pectinase test: - represents the absence of the activity. + represents <1 mm zone, ++ represents <2 mm zone, +++ represents >2 mm zone.

**Table 4:** Description of antagonistic activity. Data are mean of triplicate with SE± and alphabets represent significant different at the levels of *p* ≤ 0.05.

Treatments	Fungus Growth (cm)	Bacterial Zone (cm)	Percentage Growth Inhibition (%)
Control	0.00 ^i^ ± 0.0	0.00 ^h^ ± 0.0	0.00 ^i^ ± 0.0
MRS-3	5.16 ^c^ ± 0.01	2.16 ^d^ ± 0.01	35.67 ^f^ ± 1.01
MRP-7	3.36 ^h^ ± 0.011	2.66 ^a^ ± 0.017	58.33 ^a^ ± 1.91
MRP-8	4.26 ^f^ ± 0.012	2.06 ^e^ ± 0.015	47.33 ^c^ ± 1.54
MRS-10	0.00 ^i^ ± 0.0	0.00 ^h^ ± 0.0	0.00 ^i^ ± 0.0
MRP-12	3.56 ^g^ ± 0.01	2.46 ^b^ ± 0.012	55.67 ^b^ ± 1.23
MRS-13	0.00 ^i^ ± 0.0	0.00 ^h^ ± 0.0	0.00 ^i^ ± 0.0
MER-15	5.36 ^b^ ± 0.02	1.76 ^f^ ± 0.009	33.33 ^g^ ± 1.51
MRS-17	4.76 ^d^ ± 0.021	2.46 ^b^ ± 0.01	40.66 ^f^ ± 1.67
MRS-20	0.00 ^i^ ± 0.0	0.00 ^h^ ± 0.0	0.00 ^i^ ± 0.0
MRP-23	0.00 ^i^ ± 0.0	0.00 ^h^ ± 0.0	0.00 ^i^ ± 0.0
MRS-27	6.16 ^a^ ± 0.023	1.23 ^g^ ± 0.01	23.33 ^h^ ± 1.12
MRS-29	0.00 ^i^ ± 0.0	0.00 ^h^ ± 0.0	0.00 ^i^ ± 0.0
MRS-33	4.46 ^e^ ± 0.01	2.26 ^c^ ± 0.01	44.33 ^d^ ± 1.21
MRP-34	0.00 ^i^ ± 0.0	0.00 ^h^ ± 0.0	0.00 ^i^ ± 0.0
MRS-35	0.00 ^i^ ± 0.0	0.00 ^h^ ± 0.0	0.00 ^i^ ± 0.0

**Table 5:** Root shoot length and No. of pods and seeds in pods. Data are mean of triplicate with SE± and alphabets represent significant difference at the levels of *p* ≤ 0.05.

Treatment	Root Length (cm)	Shoot Length (cm)	No. of Pods	No. of Nodules
To-Control	8.200 ^f^ ± 0.981	14.467 ^g^ ± 0.85	2.6667 ^d^ ± 0.3	3.6667 ^d^ ± 0.2
T1-Control with fungus	5.6667 ^g^ ± 0.99	11.667 ^h^ ± 0.77	1.6667 ^d^ ± 0.0	2.0000 ^d^ ± 0.1
T2-MRP-8	15.767 ^a^ ± 1.34	24.633 ^a^ ± 0.93	10.333 ^a^ ± 0.1	11.6667 ^a^ ± 0.16
T3-MRP-12	14.500 ^b^ ± 1.51	23.633 ^b^ ± 2.01	9.667 ^a^ ± 0.1	8.6667 ^c^ ± 0.41
T4-MRP-7	11.967 ^de^ ± 1.22	20.067 ^d^ ± 1.02	8.0000 ^b^ ± 0.0	11.667 ^a^ ± 0.23
T5-MRS-33	12.667 ^cd^ ± 0.93	20.800 ^c^ ± 1.11	7.6667 ^b^ ± 0.0	9.0000 ^bc^ ± 0.11
T6-MRS-17	11.333 ^e^ ± 1.29	18.667 ^e^ ± 1.32	7.0000 ^b^ ± 0.1	11.333 ^ab^ ± 0.21
T7-MRS-3	13.167 ^c^ ± 0.21	17.867 ^f^ ± 1.71	5.6667 ^c^ ± 0.0	8.3333 ^c^ ± 0.1
T8-MIX	14.500 ^b^ ± 0.89	22.967 ^b^ ± 1.09	8.0000 ^b^ ± 0.0	12.333 ^a^ ± 0.22

## Data Availability

All the data are presented in this study.
